# Effect of Periodontal Surgery on Osteoprotegerin Levels in Gingival Crevicular Fluid, Saliva, and Gingival Tissues of Chronic Periodontitis Patients

**DOI:** 10.1155/2015/341259

**Published:** 2015-02-28

**Authors:** Sandy H. S. Hassan, Mahmoud I. El-Refai, Noha A. Ghallab, Rehab Fawzy Kasem, Olfat G. Shaker

**Affiliations:** ^1^Department of Oral Medicine, Periodontology and Oral Diagnosis, Faculty of Dentistry, Fayoum University, Fayoum 6351, Egypt; ^2^Department of Oral Medicine, Periodontology and Diagnosis, Faculty of Oral and Dental Medicine, Cairo University, Cairo 11553, Egypt; ^3^Department of Oral Pathology, Faculty of Oral and Dental Medicine, Cairo University, Cairo 11553, Egypt; ^4^Department of Medical Biochemistry, Faculty of Medicine, Cairo University, Cairo 11553, Egypt

## Abstract

*Objectives*. This study was undertaken to investigate the OPG profiles in gingival crevicular fluid (GCF), saliva, and gingival tissues of chronic periodontitis (CP) patients in response to open flap debridement (OFD). *Subjects and Methods*. The study included 30 subjects divided into 2 groups: 20 CP patients and 10 periodontally healthy subjects. Plaque index, gingival index, pocket depth, and clinical attachment level measurements were recorded for all subjects. GCF, salivary, and gingival samples were collected from all 30 subjects at baseline and 3 and 6 month after OFD from the 20 CP patients. GCF and salivary OPG levels were assessed by ELISA assay, while OPG expression in gingival tissues was examined by immunohistochemistry. *Results*. GCF, salivary and gingival OPG profiles were significantly higher in control subjects compared to CP patients at baseline (*P* < 0.001). Within CP group, OPG levels in GCF, saliva, and gingival samples showed a significant increase at 3 and 6 months after OFD (*P* < 0.001) compared to baseline. Although OPG values increased significantly in gingival samples and insignificantly in saliva after 3 months compared to 6 months, yet GCF levels were significantly decreased. *Conclusions*. OPG might be considered as a diagnostic and prognostic biomarker of periodontal bone destruction. This trial is registered with NCT02160613.

## 1. Introduction

The recognition that periodontitis involves an inflammatory component as well as altered bone metabolism has provided a new perspective on the aetiology of the disease. Investigations into the pathogenesis of periodontal disease are now considered to fall under the umbrella of “osteoimmunology” [1]. Under normal physiologic conditions, the integrity of bone tissues depends on maintaining a delicate equilibrium between bone resorption by osteoclasts and bone formation by osteoblasts which promotes bone homeostasis [1, 2]. The major regulatory mechanism of osteoclasts activity is driven by members of the TNF receptor superfamily, RANK (receptor activator of nuclear *κ*B), RANKL (RANK ligand), and osteoprotegerin (OPG). The imbalances of this system are pivotal components of the etiology of periodontal diseases [3, 4].

OPG is a novel soluble decoy receptor that has been called the “bone protector” as it protects the skeleton from excessive bone resorption [5]. The OPG cellular sources are osteoblasts, endothelial cells, fibroblasts, and vascular and smooth muscle cells [6–8]. When OPG concentrations are high, it binds to RANKL, inhibiting RANKL–RANK interaction, thus suppressing the terminal stage of osteoclastic differentiation and activation and blocking osteoclastogenesis. OPG has been also reported to induce apoptosis of mature osteoclasts [2, 9, 10]. In contrast, during an inflammatory response, inflammatory mediators enhance the expression of RANKL on periosteal osteoblasts' surface. RANKL becomes available to bind to the receptor RANK on osteoclast precursors, tipping the balance towards osteoclast activation and bone resorption [2, 4]. Whether bone resorption or bone formation occurs depends critically on the RANKL/OPG ratio, which is a function of relative expression levels of RANKL and OPG [11].

There is now a wide body of evidence in the literature indicating that alveolar bone destruction is associated with an imbalance in RANKL and OPG [12]. Previous studies reported that the expression of OPG was found to be decreased, while RANKL was increased in periodontal diseases [13–17]. Bostanci et al. [18] have shown that RANKL and OPG gene expressions are differentially regulated in gingival tissues depending on the form of periodontal disease, and increased RANKL/OPG expression ratio may indicate the occurrence of periodontitis. Another line of studies has reported similar results for OPG in gingival crevicular fluid (GCF) [19, 20] and saliva. Buduneli et al. [21] evaluated the effects of initial periodontal treatment on GCF levels of IL-17, soluble RANKL, and OPG in smoking and nonsmoking patients with chronic periodontitis. The authors reported that GCF OPG levels decreased in smokers and nonsmokers after periodontal therapy; however, RANKL levels did not differ between groups or with treatment.

A number of proinflammatory biomarkers and bone turnover-related molecules have been detected in GCF and saliva [22, 23] which emerged as possible markers of periodontal disease activity [24], thus providing a rational basis for investigating OPG in this study. Given the complex nature of periodontitis, it is unlikely that one single laboratory examination can address all issues concerning diagnosis and prognosis [25]; accordingly this study also evaluated the expression of OPG in gingival tissues. Meanwhile, there is only little information on the possible effects of periodontal therapy on OPG levels. Thus, the aim of this investigation was to estimate OPG levels in GCF, saliva, and gingival tissues of severe chronic periodontitis patients before and after open flap debridement (OFD) procedure.

## 2. Subjects and Methods

### 2.1. Study Population

Thirty systemically healthy subjects were enrolled in this study. The study included two main groups: 20 patients suffering from severe chronic periodontitis (age range 35–55 years) and 10 periodontally healthy volunteers as control subjects (age range 32 to 51 years). A detailed medical history of each subject was obtained according to the detailed questionnaire of the modified Cornell Medical Index [26]. Written consent was obtained from each subject, who signed an informed consent form approved by the research ethics committee, after explaining the study as well as giving information about the treatment and follow-up appointments needed. This clinical trial has been registered in U.S. National Institutes of Health Clinical Trials Registry, ClinicalTrials.gov, the clinical trial registration number is Identifier: NCT02160613, and name of the trial register is OPG_OFD.

### 2.2. Inclusion Criteria

Chronic periodontitis (CP) patients were selected from the Outpatient Clinic, Department of Oral Medicine, Periodontology and Diagnosis, Faculty of Oral and Dental Medicine, Cairo University, between April 2013 and June 2013. To qualify for the study, patients were diagnosed with severe generalized CP, having pocket depth (PD) of ≥5 mm and clinical attachment level (CAL) ≥5 mm, according to the American Academy of Periodontology [27]. The control group (*n* = 10) was selected from healthy subjects who attended the restorative dental clinic and had clinically healthy gingiva with zero plaque index (PI), gingival index (GI), and CAL (≤3 mm PD).

### 2.3. Exclusion Criteria

The following criteria were also used to exclude subjects in the study: (1) pregnant women; (2) subjects having <22 permanent teeth; (3) subjects having any given systemic disease; (4) subjects taking any type of medication and/or antibiotic therapy during the 3 months before the study; (5) subjects who received periodontal treatment within the past 12 months; and (6) current or former smokers.

### 2.4. Evaluation Parameters

The evaluation in this study included assessing OPG profiles in GCF, saliva, and gingival tissues for CP patients at baseline and 3 and 6 months after OFD. Control subjects had normal healthy periodontium; therefore, their OPG profiles were assessed in GCF, saliva, and gingival tissues only at the beginning of this study. Only one examiner, the same one charged with clinical measures, collected all GCF, salivary, and gingival samples.

### 2.5. Clinical Parameters

Chronic periodontitis patients received clinical examination including the following periodontal parameters: PI, GI, PD, and CAL. These measurements were recorded all by a single calibrated examiner at six sites for all teeth mesiobuccal, mesiolingual, midbuccal, distobuccal, distolingual, and midlingual. Calibration exercises for probing measurements were performed in five patients before the actual study. The intraexaminer agreement was good, with a 0.83 *κ* value. PI was established by measuring the presence or absence of supragingival biofilm with a sweeping movement of the probe around the surfaces of all teeth [28]. Marginal gingival bleeding was recorded with GI [29]. PD was measured from the free-gingival margin to the base of the periodontal pocket and CAL was measured from the cementoenamel junction to the base of the periodontal pocket. Measurements were rounded to the highest whole millimeter using the Michigan 0 probe with Williams' markings.

### 2.6. Periodontal Therapy

After initial examination, CP patients were given detailed instructions on self-performed plaque control measures using soft toothbrush and interdental cleansing devices. Full mouth supra and subgingival scaling and root planning were performed by the same operator. Subgingival debridement included use of ultrasonic devices (NSK nonoptic ultrasonic scaler, Kanuma-shi, Japan) and periodontal Gracey curettes under local anesthesia (Lustra Gracey periodontal curettes, Dentsply, Surrey, UK). The end point of mechanical treatment included removal of all subgingival calcified deposits to achieve a smooth and hard surface. For each patient reassessment was performed after 4 weeks and those patients who were presented with PD ≥ 5 mm and CAL ≥ 5 mm were scheduled for surgical procedure. An access flap was performed (OFD). In brief, full thickness flaps were elevated to fully expose the defects. Defects were fully debrided and roots were carefully planned. Flaps were repositioned and sutured to obtain primary closure of the interdental space. Patients were instructed to rinse with 0.12% chlorhexidine gluconate (Antiseptol, Kahira Pharmaceuticals Co., Egypt) oral rinse twice daily for assistance in plaque control for 2 weeks. Three weeks postsurgically the patients were instructed to gently brush the operated area with a soft tooth brush using roll technique. No interdental cleaning was attempted until one-month postsurgically.

### 2.7. GCF and Saliva Samples Collection

Following the careful removal of supragingival biofilm, areas were washed with water spray, isolated with cotton rolls, and gently dried. GCF was collected by placing filter paper strips (Periopaper, IDE Interstate, Amityville, NY, USA) into the pocket until a slight resistance was perceived and then left in place for 30 s. Filter paper strips were placed in the site with deepest periodontal pocket. Strips contaminated by blood were excluded. The strips were placed into sterile Eppendorf tubes containing 300 *μ*L PBS. All GCF samples were immediately stored at −80°C until subsequent analysis.

Collection of unstimulated whole saliva was done using standard techniques [30]. Samples were obtained by requesting subjects to swallow first, tilt their head forward, and expectorate all saliva in tube for 5 min without swallowing. After collection, all saliva samples were centrifuged at 2000 ×g and the supernatants were separated and stored at −80°C until subsequent analysis.

### 2.8. Determination of Osteoprotegerin in GCF and Saliva Samples

GCF and salivary samples were analyzed for OPG using commercially available human enzyme-linked immunosorbent assay (ELISA) kit (BioVendor Researches & Diagnostic Product European Union). Analyses were performed according to the manufacturer's protocol. All ELISA determinations were performed in duplicate. It is a sandwich-type ELISA where a monoclonal anti-human OPG, adsorbed ontomicro wells, binds to OPG in the sample, respectively. Results were calculated using the standard curves included in each assay kit. The intensity of the color was measured at 450 nm. The concentration of OPG was determined in picograms/milliliter (pg/mL).

### 2.9. Assessment of Osteoprotegerin in Gingival Tissues

#### 2.9.1. Tissue Specimens

Gingival biopsies were taken from 30 subjects participating in this study and were about 2 mm in diameter. Gingival biopsies from 20 CP patients were taken during OFD procedure as baseline samples. After 3 and 6 months 40 biopsies from the tissue inside the defect (1 mm × 1 mm) were taken and were dissected away by tip of a sharp curette without disturbing the healing phase [31]. Ten healthy gingival control samples were harvested from individuals who agreed to undergo a crown-lengthening procedure for prosthodontic purposes and whose periodontal statuses were clinically healthy. Thin (5 *μ*) paraffin sections of each tissue specimen were stained with H&E stained sections and were studied under ordinary light microscope to monitor the inflammatory changes within tissues throughout the study period. Seventy paraffin sections were mounted on positively charged slides (OptiPlus, BioGenex, USA) (10 normal, 20 at base line, 20 at 3 months, and 20 at 6 months) for immunostaining with anti-osteoprotegerin antibody. The glass slides were boxed and stored at 20°C until processed for immunohistochemical staining.

#### 2.9.2. Immunohistochemical Evaluation of OPG

The 70 paraffin embedded tissue sections on positively charged slides were immunostained with antiosteoprotegerin antibody with super sensitive biotin-streptavidin staining technique. Tissue sections were deparaffinized, rehydrated, and treated with endogenous peroxidase in 0.3% H_2_O_2_ for 30 min to block the endogenous peroxidase activity. For antigen retrieval, the slides were boiled in 10 mM citrate buffer, pH 6.0 for 10–20 min, followed by cooling at room temperature for 20 min. The positive test slides were incubated with the primary antibody rabbit polyclonal anti-osteoprotegerin antibody (cat #: AF805 with concentration 5–15 *μ*g/mL R&D system, USA) (purified IgG was used), with the appropriate dilution range 1 : 50 for 30 min at room temperature in a humidified chamber, while the negative control slides were not exposed to the primary antibody. After washing with phosphate buffer solution (PBS), the slides were treated with the biotin labeled link antibody; then the streptavidin conjugated to horseradish peroxidase was used. The diaminobenzidine chromogen was applied to visualize the antigen antibody reaction. All these reagents belong to the universal Labeled Streptavidin-Biotin 2 System, Horseradish Peroxidase (code number K0673 DakoCytomation, Denmark). All the slides were immersed in Mayer's hematoxylin for counterstaining. Finally, the sections were covered by coverslips using aqueous mounting medium.

The ordinary light microscope was used to detect and localize the osteoprotegerin immunostaining. Slides were considered to stain positively if all, or at least clusters of cells, displayed a brown dark color. Then, all the sections were examined by an image analyzer computer system using the software Leica Qwin 500 (Leica Microsystems Imaging Solutions Ltd, Cambridge, UK). Five random fields in each specimen were captured using a magnification (×400) to determine the area percentage and immunostaining intensity of the positive tumor cells. The OPG positive sections were visually scored by three observers due to the small size of the tissue biopsies. Each slide was evaluated with 2 scores on 1–3 scale for two parameters: the distribution of the OPG immunoreaction (area of stain) and the intensity of the stain [14]. The scores of both area percentage and immunostaining intensity were then summed to obtain a single total score. The overall reaction was considered mild (Scores 1 and 2), moderate (Scores 3 and 4), or strong (Scores 5 and 6) according to the single total score.

### 2.10. Statistical Analysis

Statistical power calculations indicated that ≥17 CP patients would be required when *α* was 0.05 to obtain 95% power. Mean and SD values for clinical parameters and OPG levels were calculated for all study groups. Paired *t*-test was used to study changes by time in PD, CAL, and OPG levels as they showed normal (parametric) distribution. Student's *t*-test was used to compare between CP and control groups for normal parametric distribution. Wilcoxon signed-rank test was used to study changes by time in PI, GI, and immunostain scores as they showed nonnormal (nonparametric) distribution. Mann-Whitney *U* test was used to compare between staining scores of CP and control groups. Pearson's correlation coefficient was used to determine significant correlations between OPG levels and different variables. Spearman's correlation coefficient was used to determine significant correlation between staining scores and different variables. *P* values that were less than or equal to 0.05 were considered statistically significant. All data were processed with a computerized statistical package IBM (7 Corporation, NY, USA) SPSS (8 Inc., IBM Company) Statistics Version 20 for Windows.

## 3. Results

### 3.1. Clinical Parameters

The distributions of subjects according to the age and gender are presented in Table 1. Subjects for whom clinical data were recorded included only the 20 patients diagnosed with severe CP (Table 2). The present data showed that OFD resulted in 38.5% PPD reduction and 16.7% CAL gain after 3 months which increased by the end of 6 months to reach 47.9% and 17.5%, respectively. A statistically significant decrease in all clinical parameters at 3 months and 6 months compared to baseline, as well as from 3 to 6 months (*P* ≤ 0.001), was evident. However, CAL showed no significant difference between 3 and 6 months postoperatively (*P* = 0.278).

### 3.2. Results of OPG Levels in GCF and Saliva

**Figure 1:**
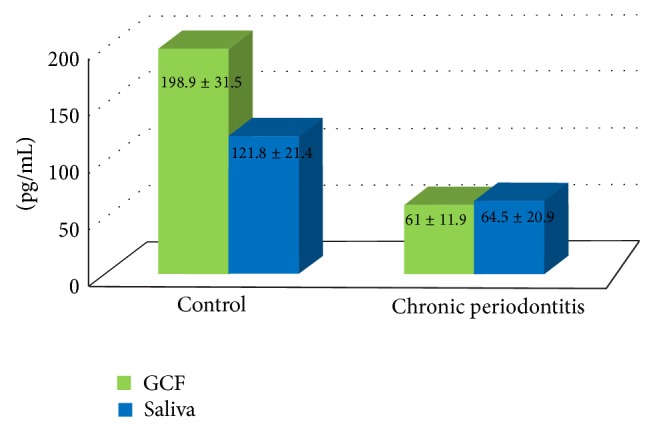
Bar chart representing mean GCF and salivary OPG levels of the two studied groups at baseline.

**Figure 2:**
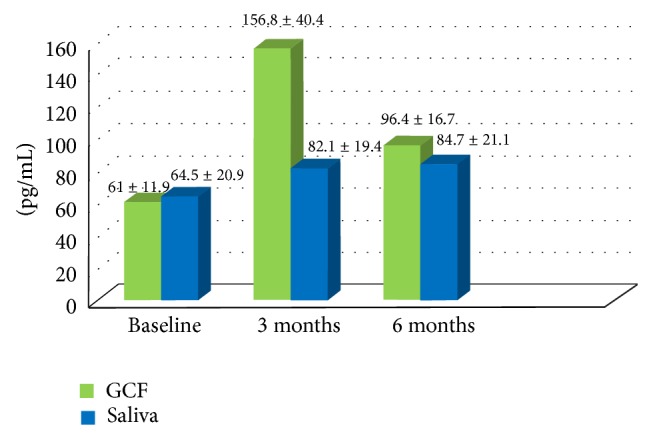
Bar chart representing mean GCF and salivary OPG levels of chronic periodontitis group throughout the experimental period.

Baseline GCF and saliva OPG profiles were assessed for all 30 subjects who participated in this study (Figure 1). These results showed that GCF (198.9 ± 31.5) and salivary (121.8 ± 21.4) OPG concentrations were significantly higher in control group (*P* < 0.001) compared to CP group at baseline (61 ± 11.9 and 64.5 ± 20.9, resp.) and 6 months postsurgical. Regarding CP patients GCF and salivary OPG profiles are shown in Figure 2 and Table 2. This study showed a statistically significant increase in GCF and salivary OPG levels at 3 and 6 months after OFD compared to baseline values (*P* < 0.001). Although salivary OPG values increased after 6 months compared to 3 months, yet this difference was not statistically significant (*P* = 0.293). In contrast, OPG-GCF values decreased significantly after 6 months compared to 3 months levels (*P* < 0.001). In CP group, no statistically significant correlation was demonstrated between GCF-OPG levels and all clinical parameters throughout the experimental period. However, the present study reported a statistically significant inverse (negative) correlation (*r* = −0.791) between salivary OPG levels and PD at baseline and between salivary OPG and PI (*r* = −0.737) after 6 months (*P* < 0.001). Yet, no statistically significant correlation was observed between salivary OPG levels and the other clinical parameters at baseline and after 6 months. Likewise, no statistically significant correlation was observed between all clinical variables and salivary OPG levels 3 months postoperatively.

### 3.3. Results of OPG Immunohistochemical Expression in Gingival Tissues

**Figure 3:**
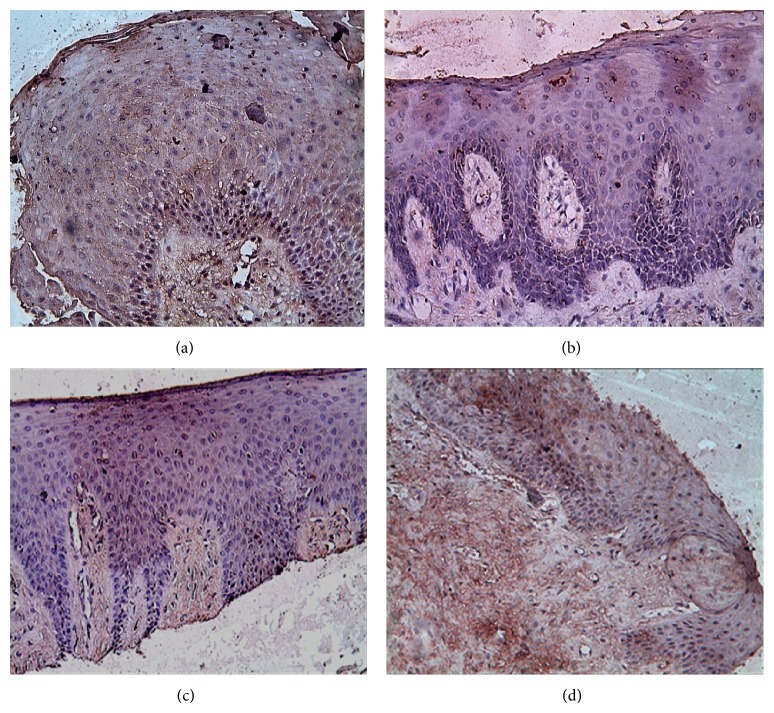
Photomicrograph of a section from gingiva of control group showing strong positive OPG immunostain within both epithelium and connective tissues in (a), photomicrograph of a section from gingiva of CP group at baseline showing weak positive OPG immunostain within both epithelium and connective tissues in (b), and photomicrograph of a section from gingiva of CP group showing moderate OPG immunostain within both epithelium and connective tissues at 3 months in (c) and at 6 months in (d) (anti-OPG antibody ×200).

The positive immunohistochemical stain within gingival sections was in form of brown membranous immunostain within the epithelial lining and diffuse positive brown stain within the connective tissue (Figure 3). This study observed that OPG staining was significantly stronger in control group tissues than tissue from sites within CP group at baseline and 6 months after OFD (*P* < 0.001). OPG staining was moderate to strong in 10/10 (100%) of control specimens while specimens obtained from CP group at baseline showed weak OPG expression 4/10 (20%) which was concentrated within the underlying connective tissue in relation to the epithelial lining. Sections obtained from CP group showed a statistically significant gradual increase in OPG immunostain within the connective tissue from 3 months (weak to moderate) 13/20 (65%) to 6 months (moderate) 17/20 (85%) which was also statistically significant (*P* < 0.001) compared to baseline scores.

Finally, this investigation demonstrated no correlation between levels of OPG in GCF, saliva, and gingival tissues when compared to each other throughout the study period.

## 4. Discussion

There is currently a wide body of convincing evidence for the role of the RANKL–OPG system in periodontitis that could be exploited for diagnostic or therapeutic purposes [12]. Molecular and immunological analyses are now essential to verify the effect of different biomarkers associated with periodontal diseases [32]. While GCF has several diagnostic advantages, yet there are some difficulties including the following: being time consuming, being exhaustive, and requiring multiple sampling of sites and sample contamination. Acquisition of saliva is easy, noninvasive, and rapid and requires less manpower and materials than GCF. Since diverse mediators involved in alveolar bone remodelling are continuously washed into saliva by GCF, hence, collection and analysis of salivary biomarkers constitutes a reliable alternative to GCF sampling [33, 34].

The current study reported statistically significant higher OPG levels in GCF, saliva, and gingival tissues of healthy subjects than chronic periodontitis (CP) patients. These results are in accordance with previous studies investigating OPG in GCF [19, 20, 35]. The increased level of OPG in saliva of CP patients compared with healthy subjects is consistent with the results shown by Tobón-Arroyave et al. [36]. According to the authors, these findings appear to correspond well with those detected in GCF samples using similar detection techniques [21]; therefore, it might be possible to hypothesize that salivary OPG originated from GCF. Tobón-Arroyave et al. [36] further explained their results by the fact that osteoclast differentiation regulation is based on the OPG expressed in the microenvironment surrounding osteoclast precursor cells [37]; thus, increased salivary OPG in CP patients might constitute a reflection of the actual biological activity of the basic multicellular unit within the periodontal ligament and alveolar bone and of the existing clinical periodontal status, thus indicating that a negative bone balance persists in periodontitis-affected tissues, which might also explain the findings presented herein.

The present findings also support previous reports examining OPG expression in the gingival tissues of patients with periodontitis [13, 14]. Bostanci et al. [18] and César-Neto et al. [38] reported lower OPG expression levels in CP in comparison to health, at a range of 0.2–16-fold. The current data showed that OPG levels in gingival tissue, GCF, and saliva are all regulated in a similar manner in periodontal disease, which denotes that OPG concentration in GCF and saliva might be an important indicator of periodontitis, as it mirrors the relative expression in the tissue. A tenable explanation for these results is that CP patients have fewer bone associated cells and less bone-surrounding tissue because of bone resorption; thus, the cells supplying OPG would also be fewer, resulting in a smaller quantity of this modulator being released into the GCF [19]. Furthermore, various proinflammatory cytokines detected in GCF from CP patients [39, 40] have been found to inhibit OPG mRNA and protein levels in osteoblasts [41, 42]. Collectively, these findings suggest that the decreased OPG levels in CP patients might constitute a reflection of the actual biological activity within the alveolar bone.

A more complex point that warrants further investigation is the association of OPG to clinical measures of periodontal disease. The current study showed that GCF-OPG concentrations were not associated with clinical measurements of disease severity (i.e., PD and CAL) and inflammation (i.e., GI). There are inconsistencies between studies, indicating that OPG correlates negatively [19], positively [20], or not at all [43] with disease severity. Still, there appears to be no correlation between OPG in GCF and gingival inflammation [19, 20]. In support with Bostanci et al. [18], this study also observed that OPG expressions in gingival tissues were not correlated with clinical parameters.

In contrast to data presented herein, Lu et al. [43] showed that OPG was inconsistently found in a few GCF samples of diseased sites and was even undetectable in any of the control sites. Such results may also account for variant levels of subclinical inflammation among healthy subjects. Regarding salivary OPG levels, Tabari et al. [44] failed to demonstrate a significant difference between healthy and CP patients while Costa et al. [45] showed that it was higher in CP and diabetic patients than in the control group suggesting that OPG concentration could increase under circumstances that require the inhibition of osteoclastogenesis. Moreover, Garlet et al. [15] observed equal prevalence of OPG expression in periodontitis patients and control, even a more intense expression was detected in the diseased group. As bone remodelling biomarkers are driven by a variety of mechanisms, a partial explanation for these discrepancies could be attributed to the differences in the sampling methods, sensitivity/specificity of the immunoassays, and intraindividual differences between study populations and sample size.

The present investigation demonstrated for the first time that OPG profiles increased significantly in GCF, saliva, and gingival tissues of CP patients after OFD compared to presurgical levels. Increased OPG concentrations after nonsurgical periodontal treatment have been previously reported in GCF of patients with diabetes mellitus [46] as well as in saliva of CP patients [47]. In addition, the later study demonstrated increased salivary OPG levels after treatment by oral hygiene instructions only and hypothesized that OPG reflects improved periodontal health as a result of localized therapy. The authors also showed significant correlation between all clinical parameters and salivary OPG levels. The former results are in line with the current study which showed a significant inverse correlation between salivary OPG levels and baseline PD as well as between salivary OPG levels and PI after OFD. These findings further strengthen the diagnostic value of OPG profiles, indicating that the biomarker shows some specificity for the occurrence of periodontal bone destruction rather than constituting a “conventional” marker of periodontal inflammation. Although insignificant, Bostanci et al. [48] also observed an increase in GCF-OPG levels in CP patients after scaling and root planning. Consistent with the present data, Buduneli et al. [49] showed higher levels of salivary OPG in treated periodontitis patients in the maintenance phase than that in untreated periodontitis patients. Increased OPG may be indicative of a reduction in the inflammatory process which denotes that removal of the inflammatory stimulus and maintaining a level of plaque control have a beneficial effect. Interestingly, this research observed that GCF-OPG levels in CP patients decreased from 3 to 6 months after periodontal surgery. These findings denote that the molecular mechanisms of bone resorption are still active, and thus the corresponding periodontal sites can be potentially at a risk of further disease relapse. Accordingly, this study highlights the importance of the supportive periodontal therapy in order to guarantee optimal long-term prognosis.

On the contrary, previous studies [21, 46, 50, 51] assumed that a clinically successful nonsurgical treatment outcome may not predictably result in reducing OPG levels. It has been hypothesized that the inflammatory process in the GCF sampling sites may have been inactive at baseline when they took the samples. This discrepancy might also be attributable to the tissue healing, since conventional therapy alone cannot eliminate the molecular mechanisms of bone resorption and thus a risk for further periodontal tissue breakdown may still exist. Dereka et al. [50] estimated OPG expression in gingival tissues from healthy and periodontally affected patients 4–6 weeks after nonsurgical periodontal therapy. The authors demonstrated that OPG mRNA was more expressed in healthy samples compared to samples from treated CP patients; however, immunohistochemical analysis showed that OPG levels were higher in CP compared to healthy specimens. As explained by the authors, the probable differences in the pathological process among the periodontitis patients group and the time period between nonsurgical periodontal treatment and surgical procedure might reveal a various healing pattern in these patients [15].

## 5. Conclusion

The current study showed decreased OPG levels in GCF, saliva, and tissue of CP patients compared to healthy controls which provides further evidence regarding the role of OPG downregulation in periodontal disease. This investigation, to the best of our knowledge, demonstrates for the first time increased levels of OPG in GCF, saliva, and gingival tissues of CP patients after periodontal surgery. These results might indicate the possible involvement of OPG in the regulation of periodontal tissue repair suggesting a diagnostic and prognostic potential of this biomarker in periodontal disease. Although larger samples and longitudinal studies are required to confirm this potential, the research opens the door to explore OPG as a promising new treatment strategy for inhibiting periodontal bone destruction.

## Figures and Tables

**Table 1:** Sample description.

Group	Chronic periodontitis	Control
Number of subjects	20	10
Age (years; mean ± SD)	41.2 ± 4.8	37.81 ± 8.3
Gender (*n*; female/male)	12/8	6/4

SD: standard deviation.

**Table 2:** The mean and standard deviation of different variables estimated in chronic periodontitis patients throughout the experimental period.

Time	Parameter					
	GCF-OPG (pg/mL)	Salivary-OPG (pg/mL)	PI	GI	mm PD	mm CAL
Baseline	61 ± 11.9	64.5 ± 20.9	2.01 ± 0.46	1.87 ± 0.37	5.39 ± 0.56	6.2 ± 0.77
3 months	156.8 ± 40.4^*^	82.1 ± 19.4^*^	0.62 ± 0.25^*^	0.64 ± 0.35^*^	3.29 ± 0.36^*^	5.21 ± 0.79^*^
6 months	96.4 ± 16.7^∗#^	84.7 ± 21.1^*^	0.33 ± 0.13^∗#^	0.41 ± 0.23^∗#^	2.78 ± 0.32^∗#^	5.17 ± 0.83^*^

^*^Statistically significant difference from the baseline.

^#^Statistically significant difference from the 3 months.
